# Anti-saccade as a Tool to Evaluate Neurocognitive Impairment in Alcohol Use Disorder

**DOI:** 10.3389/fpsyt.2022.823848

**Published:** 2022-04-27

**Authors:** Yuqi Si, Lihui Wang, Min Zhao

**Affiliations:** ^1^Shanghai Mental Health Center, Shanghai Jiao Tong University School of Medicine, Shanghai, China; ^2^Shanghai Key Laboratory of Psychotic Disorders, Shanghai Mental Health Center, Shanghai Jiao Tong University School of Medicine, Shanghai, China; ^3^Institute of Psychology and Behavioral Science, Shanghai Jiao Tong University, Shanghai, China; ^4^Shanghai Key Laboratory of Psychotic Disorders, Shanghai, China; ^5^CAS Center for Excellence in Brain Science and Intelligence Technology (CEBSIT), Chinese Academy of Sciences, Shanghai, China

**Keywords:** alcohol use disorder, anti-saccade, cognitive impairment, attention bias, inhibitory control

## Abstract

It has been widely shown that chronic alcohol use leads to cognitive dysfunctions, especially inhibitory control. In an extension of the traditional approach, this research field has benefited from the emergence of innovative measures, among which is an anti-saccade, allowing direct and sensitive measure of the eye movements indexing attention bias to alcohol-related cues and the capability of inhibiting the reflexive saccades to the cues. During the past decade, there are numerous reports showing that drinkers make more unwanted reflexive saccades and longer latency in the anti-saccade task. These increased errors are usually explained by the deficits in inhibitory control. It has been demonstrated that inhibitory control on eye movement may be one of the earliest biomarkers of the onset of alcohol-related cognitive impairments. This review summarizes how an anti-saccade task can be used as a tool to investigate and assess the cognitive dysfunctions and the early detection of relapsing risk of alcohol dependence.

## Introduction

Alcohol use has increased rapidly in many countries in the past decade, especially during times of the coronavirus disease 2019 (COVID-19) pandemic, due to the increase in illegal production of alcohol and the disruptions in access to medical care for alcohol dependence (1–3). A substantial body of evidence suggests that chronic excessive alcohol use can cause deficits in a broad range of cognitive functions, including memory, learning, psychomotor speed, visuospatial functioning, attention, executive functioning, and impulsivity (4). A common cognitive mechanism underlying these deficits, alcohol use disorder (AUD), may be the inflexibility of inhibitory control that can be probed with behavioral paradigms such as Stroop task and go/no-go task (5–7).

In the above-mentioned behavioral paradigms, the impaired inhibitory control on the response or attention bias to the substance-related cue was often indexed by lowered accuracy or delayed responses to complete the cognitive task when the cue was presented as distracting information. For example, conflict between task-irrelevant words and task-relevant colors in the Stroop task led to interference on the behavioral response that is required by the task. It can be tested that, relative to neutral cues, drug-related cues induce attention in drug users. The attention bias to drug-related stimuli is reflected by the increase in reaction times (RTs). However, the evidence is indirect and the effect is somewhat circumstantial because the increased RT is affected by complex cognitive processing as well as linguistic and motor abilities. The assessment of attention bias would be benefitted from multiple dimensions: What was noticed first? How long did it take the subject to attend the target? Was the attention bias due to the difficulty of disengaging attention from the drug-related cue? Addressing these issues thus asks for a direct and multidimensional index and real-time monitoring for attention bias, i.e., eye movement.

The anti-saccade task, first developed by Peter Hallet in 1978, has emerged as an imperative tool for investigating the capacity of suppressing the prepotent response (8). A correct anti-saccade performance consists of two main saccadic processes, namely, restraining a reactive saccade toward the target and then producing a saccade in the opposite direction. In this task, a sudden-onset target appears in the peripheral visual field and participants are instructed to suppress the automatic response (pro-saccade) and instead direct their gaze in the opposite direction (anti-saccade). To carry out volitional saccade in the opposite direction, the powerful urge to make a reflexive response to a sudden-onset target has to be suppressed. This effortful process results in slower latencies for correct anti-saccades and more errors than for pro-saccades.

**Figure 1:**
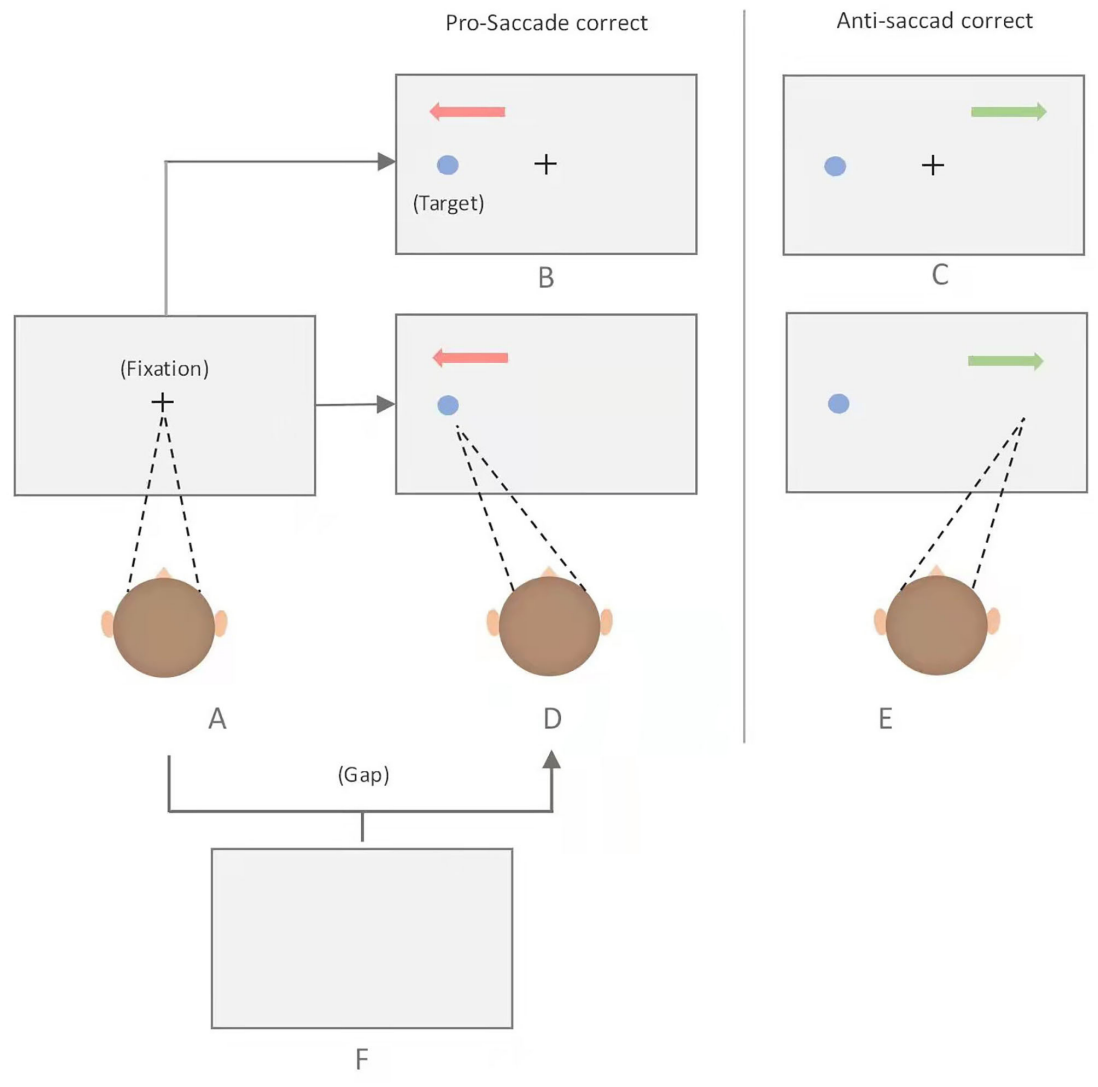
Schematic of the pro- and anti-saccade tasks. Each trial began with the presentation of a fixation point at the center of the screen, which participants are required to fixate, and to make either a prosaccade or antisaccade depending on the task rule. Immediate pro-saccade task: A-D; Immediate anti-saccase task: A-E; Gap pro-saccade task: A-F-D; Gap ant-saccade task: A-F-E; overlap pro-saccase task: A-B; Overlap anti-saccade task: A-C.

There are three variants of anti-saccade tasks, namely, gap, overlap, and immediate condition (9). The division of the three paradigms is based on the temporal difference between the disappearance of the central fixation point and the emergence of the target. If the central fixation is still there when the target appears, it is the overlap condition; if the central fixation point has disappeared for a period of time when the target appears, it is the gap paradigm; and if the central fixation just disappears when the target appears, it is the immediate condition (Figure 1). It has been suggested that anti-saccade task provides abundant sources of data to study the link among inhibitory control, executive function, and the underlying neural substrates.

In this review, first, we briefly described the neurocognitive impairment in AUD, the potential neurobiological, and neurochemical mechanisms by which alcohol consumption leads to cognitive dysfunctions. Second, we also discussed how the anti-saccade task can be used as an important tool to investigate the capability of suppressing the impulse to the substance-related stimuli in AUD, and how anti-saccade performance can predict the long-term cognitive dysfunctions and the early detection of relapsing risk of AUD.

## The Most Common Measurements in the Anti-Saccade Task

The commonly used indicators in anti-saccade tasks are as follows (10, 11): (1) saccade latency, also known as RT of the first saccade, refers to the interval from the appearance of the target to the first saccade. In the anti-saccade task, there are correct anti-saccade latency and wrong pro-saccade latency. (2) The wrong rate of saccade direction refers to the proportion of saccades made in the wrong direction in the anti-saccade task. The stimulus suddenly appearing in the visual field induces reflective saccade toward the target, which are otherwise required to be inhibited. Therefore, the direction error rate of saccade can reflect the autonomous control ability and is an important index to investigate inhibition function. In addition, saccade amplitude, velocity, and peak velocity of reverse saccade are also the basic indicators of the anti-saccade task, which can reflect the ability to execute and inhibition control.

## Neural Mechanisms of Anti-Saccades

The neural mechanisms of anti-saccades have been extensively studied, and the brain regions responsible for anti-saccades have been identified. In anti-saccade tasks, the automatic saccade (pro-saccade) and the voluntary saccade (anti-saccade) were thought to be in the planned state at the same time. Meanwhile, they have a competitive relationship in the neural activation or inhibition. The outcome of the competition ultimately determines which kind of saccade would be implemented.

Whether or not a correct anti-saccade can be generated depends on the relative activation of the neural system toward a pro-saccade and anti-saccade (12). The competition theory suggests that if the neural system performing anti-saccade reaches the activation threshold first, a correct anti-saccade will be generated and the reflexive saccade will be suppressed (13). Conversely, if the neural system performing reflexive saccade first reaches the activation threshold, it will lead to a false saccade toward the target, and then a corrected saccade may be generated. The process of the anti-saccade is more complex, and its activation threshold is relatively higher than that of a pro-saccade (14). The neural activation of the automatic saccade has to be decreased so that the anti-saccade system can reach the activation threshold.

The superior colliculus (SC) and the frontal eye fields (FEFs) contain distinct populations of fixation and saccade neurons, which play a regulatory role in the production of a correct anti-saccade through modulating discharges in a reciprocal manner (15). Compared with prosaccade trials, activity of fixation neurons is enhanced on anti-saccade trials, which can explain the anti-effect: longer RTs in anti-saccade trials than in pro-saccade trials. The contralateral dorsolateral prefrontal cortex (DLPFC) and anterior cingulate cortex (ACC) can inhibit reflexive saccade by inhibiting the neurons (16). Recently, converging evidence in experimental animals and human has revealed the neural networks that are involved in the production of anti-saccades. Clinical studies found that patients with lesions of the DLPFC struggled to perform the anti-saccade task, leading to the suggestion that the frontal lobes played a significant role in the inhibitory control (17). Similarly, by combining iontophoretic application of the general muscarinic receptor antagonist scopolamine with single-cell recordings, Major AJ et al. demonstrated that the blockade of muscarinic receptors in DLPFC led to deficits in saccade and visual direction selectivity measures (18). Johnston, Kevin et al. found that the DLPFC neurons send signals selective for stimulus location, saccade direction, and task directly to the SC through the technique of implanting electrodes in DLPFC and SC (19). These studies have provided evidence that the DLPFC may provide top-down signals to the SC neurons to inhibit the automatic saccade (20). Another area that might also send a supplementary signal to SC and FEF for the motor command of anti-saccades is the supplementary eye fields (SEFs). These commands sent to the brainstem premotor circuit can augment motor commands from the FEF and SC leading to the successful production of volitional anti-saccades (21).

It is more likely that a wrong-directional saccade and correct anti-saccade are not a series of processing processes but rather may be processed simultaneously. The individuals plan the correct anti-saccade while restraining the automatic pro-saccade. There is a certain connection between the parietal lobe region of the brain and the spatial calculation of an anti-saccade. The tasks require the perceptual motor conversion function to transform the visual stimulus into the appropriate motor command for the execution of saccades of the parietal lobe. The conversion is fast and the exact mechanism is unknown. However, evidence has accumulated to the lateral intraparietal area (LIP), and FEF might have a crucial role in vector inversion (22).

### Anti-saccades Research in Neurological and Psychiatric Disorders

**Figure 2:**
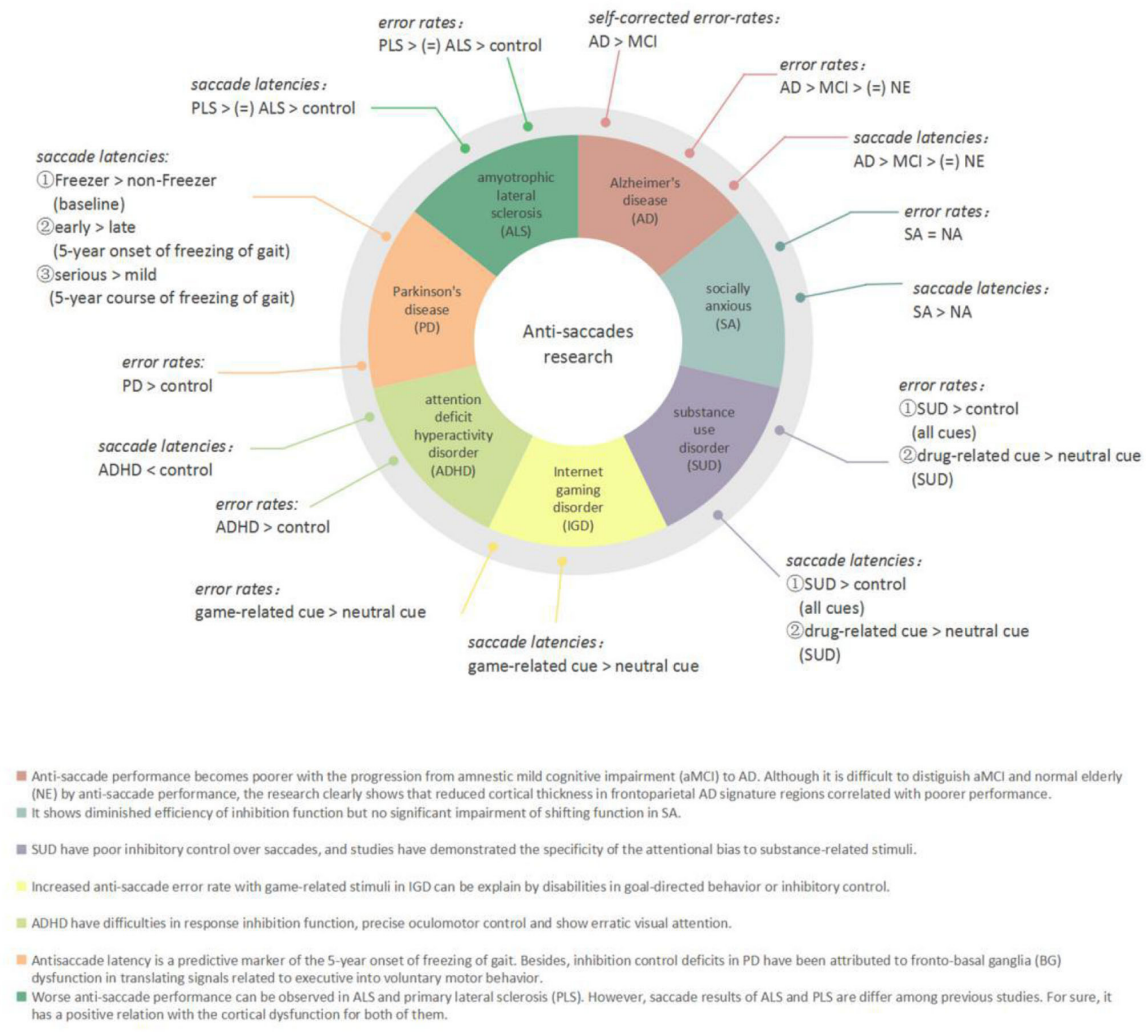
Anti-saccades research in neurological and psychiatric disorders.

Eye movement plays a vital role in the acquisition of visual information. Eye movement tasks, especially saccadic tasks, have been widely used in patients with neurological and psychiatric disorders to assess cognitive function in recent years (Figure 2). Holden et al. believed that it was a dynamic process for patients to develop from amnestic mild cognitive impairment (aMCI) to Alzheimer's disease (AD). According to this point of view, they conducted anti-saccade tasks among three groups (i.e., aMCI, mild AD, and controls), which found that patients with aMCI committed significantly more anti-saccade errors (46.9%) compared with those of controls (24.3%). Moreover, the AD group had a significantly larger self-corrected error rate than the other two groups (23). The anti-saccade task is especially useful for detecting dysfunction in selective attention in aMCI and AD (24). In some other studies, participants with AD showed reduced activation in the FEFs and posterior cortex and increased inhibitory errors when performing the anti-saccade tasks in a functional magnetic resonance imaging (fMRI) study (25). Executive dysfunction can be tested in Parkinson's disease (PD); Gallea C et al. investigated the predictive factor of freezing in PD by anti-saccade tasks and reported that compared with the non-Freezer group, the Freezer group showed equivalent motor or cognitive signs but increased anti-saccade latency before the impairment of motor or other cognitive function like memory. They concluded that anti-saccade latency is a predictive marker of the 5-year onset of freezing of gait (26). Moreover, inhibition control deficits in PD have been attributed to fronto-basal ganglia (BG) dysfunction in translating signals into voluntary motor behavior (27–29). Saccadic data also confirmed executive dysfunction in amyotrophic lateral sclerosis (ALS) as the higher percentage of direction errors in the anti-saccade tasks and increased saccadic latency (30).

Anti-saccade tasks are also used in psychiatric disorders to assess inhibitory control and explore the potential neurobiological mechanisms of the diseases. Children with attention deficit hyperactivity disorder (ADHD) exhibited shorter latency and significantly a higher anti-saccade error rate than the control group, which confirmed that children with ADHD have difficulties on precise oculomotor control and oculomotor response inhibition function (31). This kind of oculomotor control dysfunction is reflected in patients with internet gaming disorder (IGD) and socially anxious (SA). The IGD group exhibited higher saccade-error rates in the case of game-related images than in neutral or scrambled images (32). However, SA participants had longer anti-saccade latencies than non-anxious (NA) participants (33).

## Neurocognitive Impairment in AUD

### Alcohol Effects on Cognitive Function

Alcohol consumption can lead to impaired cognitive functions, accompanied with an impulse to substance-associated stimuli (34). The ability of inhibiting an automatic impulse can be indexed by the Stroop effect that assessed with the computerized version of the Stroop Color and Word Test (SCWT) (35). It is widely believed that the Stroop test is one of the gold standards of attention measures and one of the most popularly used instruments in clinical and experimental neuropsychological settings (36). In the SCWT, participants are often presented with colored color words (e.g., the word “red” printed in green) and are required to suppress the dominant tendency to read the word and instead identify the print color of the word, e.g., green) (37). The extent to which the print color is correctly and quickly identified is taken as how well the distracting word is inhibited. It has been found that patients with AUD show longer RTs in color-naming words and higher error rates on alcohol-related Stroop pictures (i.e., photos of alcoholic drinks or persons drinking alcohol) than neutral Stroop pictures (i.e., photos of kitchen items or persons in kitchen scenes), indicating an attention bias to alcohol cues relative to neutral cues (38–40). In addition, Christiansen and his colleagues showed that including individualized stimuli (e.g., the current participant's favorite drink) as the Stroop stimuli is more sensitive to measure the attention bias than including standardized stimuli (41). Studies using different paradigms, such as go/no-go tasks (42–44), visual conjunction search (VCS) tasks (45), cued visual probe tasks (cVPT) (46), and flanker tasks (47) and have consistently demonstrated that participants with AUD showed attention bias to alcohol-related cue. Moreover, these experiments also indicated that the poorer behavioral control was related to higher alcohol consumption.

In addition to attention bias and impaired executive function, AUD also showed other cognitive deficits. For instance, individuals drinking more frequently showed worse performance in a visuospatial functioning task, and this effect did not depend on more alcohol use days (48). By examining changes in working memory performance as measured by Trail Making Test-B, Lechner et al. highlighted that the function between greater alcohol-induced working memory decline and adverse consequences was mediated by the amount of the drinks per drinking day (49). However, Nguyen-Louie et al. drew a contrary conclusion about the link between adolescent alcohol with neuropsychological measurements, showing that more alcohol use days led to more neurotoxic events, and predicted worse verbal memory, visuospatial ability, and psychomotor speed, whereas unexpectedly, predicted better working memory performance (50).

### Alcohol Effects on the Brain Structure

The long-term, heavy consumption of alcohol results in reversible or irreversible modification of selective neural systems of the brain function and structure (51, 52). Brain structure damage in chronic alcoholism is well documented (53). Based on modern neuroimaging technology, an increasing number of studies have shown brain structure damage that can be related to alcohol dependence.

For example, Cosa et al. found robust magnetic resonance imaging (MRI) markers that are affected by alcohol, which showed the sensitivity and selectivity even after the relative short exposure period of alcohol consumption (54). Many animal and human models consistently demonstrated that the prefrontal cortex (PFC) is affected by chronic alcohol consumption. Extensive evidence from neuroimaging studies indicates that alcohol exposure leads to the degradation of circuitry originating from the PFC, which plays an essential role in PFC-dependent behavioral deficits in AUD (55). By using fMRI, Kreusch et al. found greater activation of PFC, in the presence of alcohol-related cues compared with neutral cues in young heavy drinkers (HDs). A potential vicious cycle was suggested that a late maturing of PFC can also increase the vulnerability of developing substance use disorder later in life including AUD (56). The DLPFC, known for its importance in cognitive function, is thought to be impaired globally by alcohol consumption.

To date, an increasing number of studies have addressed anti-saccade performance in AUD. The various forms of behavioral change in AUD show a striking similarity to that of patients with prefrontal lesions, such as AD (57). Hence, anti-saccade task performance is related to structural alterations in the frontal lobe and can be a reliable and valid biomarker for the diagnosis of AUD (58, 59).

## Anti-Saccade Research in Alcohol Use Disorder

### Dose Correlates

The impairment of impulse inhibition in AUD has been shown in previous studies using anti-saccade tasks. Specifically, a decreased anti-saccade accuracy and a specific alcohol-induced impairment in saccade amplitudes were observed in AUD (60–62). Roche DJ et al. investigated how the doses of alcohol consumption affect the eye-movement control. A total of 138 non-alcoholic social drinkers, with self-reported positive (FH+) or negative (FH–) family history, were enrolled in this study. They were divided into two groups [i.e., HDs and light drinkers (LDs)] according to the guidelines from The Substance Abuse and Mental Health Services Administration (SAMHSA). Both groups received eye-movement tests of smooth pursuit, prosaccadic, and antisaccadic task at baseline, 60 min (T1), and 180 min (T2) after the initiation of beverage consumption (63). All the subjects consumed a high alcohol dose (0.8 g/kg), a low alcohol dose (0.4 g/kg), or a beverage containing placebo (1% ethanol as taste mask) with the order randomized across separate sessions. In the anti-saccade tasks, both high dose of alcohol (0.8 g/kg) and low dose of alcohol (0.4 g/kg) increased the latency and decreased anti-saccade velocity for both HD and LD, whereas the effect of high dose was greater and more lasting. Interestingly, HDs reported less perceived deficits from alcohol than LDs in anti-saccade tasks.

It is generally considered that heavier drinkers display reduced reactions to a dose of alcohol. The theory of behavioral tolerance to alcohol posits that more experience with drinking to intoxication leads to less impaired cognitive and psychomotor performance (64). One possible explanation is that exposure to heavy or binge drinking may produce behavioral tolerance to the response to alcohol (e.g., greater motor skills). However, some research explained that behavioral tolerance in heavy drinkers occurs only at the early phase, but the heavy drinkers remain at risk in alcohol harm as they continue to engage in chronic binge drinking over time (65, 66).

### Comorbidities Correlates

Alcohol use disorder often shows attention bias to alcohol images, as revealed by eye-tracking tasks (67). The seek for alcohol significantly develops an attention bias, which has been demonstrated in previous studies (68). This effect also can be observed in the populations with co-morbid AUD and other impulse control disorders. Some research has measured the performance in these patients, and the underlying connection between AUD and the comorbidities could be probed with anti-saccade tasks.

Patients with binge eating disorder (BED) show dysfunction of inhibition on response to food cues in anti-saccade tasks, and they usually have co morbidity with AUD and meanwhile show strong impulse to alcohol cues (69–71). Drinkers may have difficulties in inhibiting saccades toward appetitive stimuli, particularly when stimuli were in the peripheral visual field (72). To assess the difference between the deficits of response inhibition between the two disorders, Schag, Kathrin et al. compared the performance between BED, AUD, and their age- and sex-matched control groups in the modified anti-saccade tasks (73). In the tasks, the stimulus in each trial was specific to the disorder (food stimuli for BED and alcohol stimuli for AUD). The results showed that BEDs made more directional mistakes in both first saccades and second saccades, which indicated that BEDs have more difficulties in inhibiting the response to stimuli regardless of the stimulus category. However, AUDs showed no discrepancy in the error rates, compared with the control groups. In addition, AUDs also performed significant less fixations to alcohol stimuli and shorter dwell times on both alcohol stimuli and neutral stimuli in free exploration tasks. Taking the two tasks into consideration, we can suggest that AUD avoid stimuli deliberately. One of the limitations of the research was that AUDs were told to be abstinent before the tasks, so that the tendency to avoid alcohol stimuli can be explained by learning efficiency. Peña-Oliver et al. investigated the diverse degrees of impulsivity to food incentives and substantiated a remarkable increase of goal-directed behavior to food incentives in alcohol-preferring rats, which might be related to their intense preference for alcohol (74). Moderate drinking can increase subsequent food intake, particularly of high-fat savory food as well (75). Christiansen P et al. investigated the effects of alcohol on energy intake and found that individuals who were given an alcohol prime (i.e., alcoholic drink mixed with diet lemonade) (0.6 g/kg) performed worse on the Stroop test and consumed more cookie calories than individuals who were given the placebo drink (76). However, the increase in energy intake was not observed in acute alcohol consumption (0.4 g/kg) in another study (77). Recent research demonstrated that alcohol consumption can increase food intake and food reward, but such effects occur only at a higher dose (0.6 g/kg) (78). A conclusion that can be drawn is that, in anti-saccade tasks, patients with BED show unselected attention bias to food cues (including alcohol-related stimuli), whereas only patients with AUD with low dose of alcohol show highly selective attention bias to alcohol-related cue. The attention bias to alcohol-related stimuli in AUD may generalize into food cues when the patients consume more alcohol.

In another line, researchers have examined the dysfunction of impulse control in patients who had comorbid alcohol and cocaine use disorder. They concluded that the anti-saccade performance could be a predictor of decreased white matter integrity (79). Given that anti-saccade performance affected by alcohol intoxication has been shown in different forms of comorbidity with AUD, the task may have the ecological validity in the way that it can be a promising tool to assess the risk of further developing into other impulse control disorder with the long-term alcohol consumption.

### Alcohol Intoxication

The development of AUD involves chronic heavy drinking to high levels of alcohol intoxication. Likewise, earlier occurred alcohol intoxication in adolescent is related to the higher rate of diagnosing AUD in the adulthood (80, 81). In addition to long-term neurocognitive impairment, some research has focused on the deficit of inhibition control during intoxication period with behavioral paradigm. In a study using the Stroop task, 20 healthy social drinkers, who reported imbibing 1–3 times a week as light-to-moderate drinking pattern and 1.5–3.5 drinks an occasion, participated in both alcohol (vodka as 20% v/v in orange juice) and placebo (the same volume of orange juice) sessions as their own control condition. The modified four-color Stroop task required individuals to respond to the color. Each word's color was consistent or inconsistent with the word meaning. There were also trials with a word in gray color, and the participants had to respond to the word meaning. Blood oxygen level-dependent (BOLD) signal was acquired using fMRI during the task. The results showed that alcohol increased RTs error rates in incongruent trials. Moderate alcohol inebriation attenuated ACC activation during both incongruent trials and erroneous responses. These findings suggested that alcohol consumption interferes with goal-directed behavior, resulting in poor inhibition control.

The Stroop evidence based on the behavior results is somewhat indirect. The cognitive processes include in such tasks involve both manual inhibition and saccade inhibition. Alcohol can also affect the motoric execution, such as the button press (82).

Anne Eileen Campbell et al. investigated the effect of alcohol on response control separately. In their study, 40 participants reported consuming 48 g ethanol in one session on at least 6 occasions in the past year, and they were required to complete each experimental task in sessions before and after drink manipulation in the alcohol testing day (i.e., 40% volume vodka in 500 ml orange juice) and placebo testing day (i.e., the same volume of orange juice with a few drops of vodka). The task can be divided into two parts, namely, manual and saccadic stop-signal reaction time (SSRT) task (83). In manual SSRT tasks, participant are told to make rapid responses with right or left index fingers to the location of the target. Similarly in saccadic SSRT tasks, saccades are produced corresponded the left and right targets. In addition, a red fixation point appears in the center of the screen as a distraction in 25% trials, and participants completed these trials with the instruction to ignore the signal and continue to make a correct button press or saccade response. The results showed that the RT (i.e., latency) reflecting manual inhibition increased during alcohol intoxication, but the RT (i.e., latency) reflecting saccadic inhibition did not increase. Their finding is different from the previous one, which noted significant increase of saccade latency by alcohol and decrease of peak velocity in classical anti-saccade tasks (61). However, both have found that saccadic error rates were not influenced by alcohol intoxication.

Below, we explained what accounts for the discrepancy in the results between the two experiments. First of all, Anne Eileen Campbell et al. employed the saccadic SSRT task in which reflexive eye movements toward sudden-onset distractive fixation point has to be inhibited but in the absence of the requirement to perform an immediate anti-saccade, which involved vector inversion and voluntary movement. Moreover, the research based on the classical anti-saccade task also detected the impairment of saccade amplitude (accurate mirrored saccadic location) under alcohol. Combined with the above results, it suggests that alcohol intoxication impairs the process of vector inversion and voluntary movement in anti-saccade but not the trigger to inhibit the flexible saccade. Second, in the saccadic SSRT task, the peripheral target appeared 12° from center (6° in anti-saccade task). Nevertheless, the latency of the corrective anti-saccade decreased with increasing stimulus distance (84). This may explain why there was no increase of saccade latency in saccadic SSRT task.

It seems that saccadic inhibition is immune to alcohol intoxication. It has been shown that the frontal cortex and SC are considered to be the key regions and command producers of saccade, respectively. On account of previous studies on the neural mechanisms of antisaccades, the immunological effect probably was due to the interactive regulatory unbalance of fixation and saccade neurons in FEF and SC (15). The saccade neurons (active in prosaccade) are inhibited more intensively than fixation neurons affected by alcohol intoxication so that the visual response that is initiated by the appearance of the target will not exceed reflexive saccadic threshold (20). However, exposing to ethanol has an effect of suppressive cortical visual event-related potentials (ERPs), which then interferes the assessment of inhibitory function in the frontal cortex. Research based on fMRIs results also demonstrated alcohol-induced activity decreases to voluntary anti-saccades and responses to erroneous responses in dorsal ACC, an area that is the central node in a predominantly frontal cortical network sub-serving inhibitory tasks (85).

## Limitations and Future Directions

Although a large number of studies have used anti-saccade tasks, these studies are not uniform in experimental settings. For example, the target eccentricity used in many studies ranges from 4° to 10°, which makes saccade latencies and error rates of different studies inappropriate to compare with each other. The history of alcohol consumption is also a significant factor in the analysis of the ethanol effects on PFC and SC. Thus, it is not conclusive to summarize the extent of impairment of inhibition control among AUDs. In addition, saccade latency and saccade error rate are frequently used in assessing AUDs, but there are relatively few studies on saccade spatial accuracy and the underlying mechanisms. This is also the content to be paid attention to in future research. In preparation for our manuscript, we have searched the databases PubMed, Cochrane library, and Web of Science up to and including October 2021 to collect the literature. Our search terms included alcohol use disorder OR alcohol dependence OR alcoholism OR alcohol intoxication AND anti-saccade OR saccade OR eye-tracking with no search limits applied. However, the relevant literature is not enough for a systematic review. First, these studies involved a wide range of subjects including abstainers, social drinkers, and alcohol intoxication. It is difficult to compare their impaired performance in anti-saccade task given the major discrepancy of inclusion criteria. Second, the different eccentricity of target stimulus could also make a significant impact on the results based on anti-saccade tasks. Finally, according to previous research, the alcohol-related stimuli could lead to an obvious deficit of saccadic inhibition control in AUD compared with the neutral stimuli. Nevertheless, the studies we searched adopted different types of cue in the tasks and assessed different ability of inhibition control (i.e., manual or saccadic), which are not appropriate for conducting a systematic analysis. Considering all things, we still think it is necessary to summarize these studies to remind the readers that the anti-saccade task is multidimensional, objective, and convenient, as a tool to assess the attention bias and inhibition control in AUD. Moreover, this is our objective to write the review, and we all hope to lay a foundation of diversified research method in AUD in the future.

In summary, as prolonged alcohol abuse is known to impair response inhibition, alcohol abusers may benefit from interventions that improve response inhibition, thereby restoring inhibitory control over automatic impulses (43, 86). Eye-movement tasks, especially the anti-saccade task, may be favorable for detecting the early impairment of inhibitory control and the risk of AD. Some new techniques such as virtual reality (VR) technology and mobile devices can be applied to assess saccadic eye movements in situations more similar to the real world (87, 88). A growing consensus that AUD and AD share common disease pathophysiology is the dysfunction in the cholinergic system in the brain, which probably can explain the preclinical abnormity showed in anti-saccade tasks (89, 90). According to this, the acetylcholine esterase (AchE) inhibitors may improve the frontal executive function in AUDs. Clinicians should also be cautious about the exposure of the social drinkers to the different types of alcohol-related stimuli during anti-saccade tasks, such as alcohol-related auditory cues (91).

These findings, taken together, indicate a new direction in future work. Aided by the early detection of inhibitory error rates in the anti-saccade task, there is increased potential for commencing effective interventions earlier for AUDs.

## Author Contributions

YS collected the data and wrote the initial draft of the review. LW and MZ did the analyses and revised the manuscript. All authors contributed to the article and approved the submitted version.

## Funding

This study was supported by the National Nature Science Foundation (81771436 and 82130041), Shanghai Municipal Science and Technology Major Project (2018SHZDZX05), Shanghai Shenkang Hospital Development Center (SHDC2020CR3045B), Shanghai Clinical Research Center for Mental Health (19MC1911100), Shanghai Engineering Research Center of Intelligent Addiction Treatment and Rehabilitation (19DZ2255200), Shanghai Key Laboratory of Psychotic Disorders (13DZ2260500), the National Science Foundation of China (No. 32000779), and the Shanghai Sailing Program (No. 20YF1422100).

## Conflict of Interest

The authors declare that the research was conducted in the absence of any commercial or financial relationships that could be construed as a potential conflict of interest. The reviewer L-ZY declared a shared affiliation with the author MZ at the time of review.

## Publisher's Note

All claims expressed in this article are solely those of the authors and do not necessarily represent those of their affiliated organizations, or those of the publisher, the editors and the reviewers. Any product that may be evaluated in this article, or claim that may be made by its manufacturer, is not guaranteed or endorsed by the publisher.
